# Characterization of odor-evoked neural activity in the olfactory peduncle

**DOI:** 10.1016/j.ibror.2020.07.010

**Published:** 2020-07-22

**Authors:** Graham A. Cousens

**Affiliations:** Department of Psychology and Neuroscience Program, Drew University, 36 Madison Avenue, Madison, NJ, 07940, USA

**Keywords:** AON, Anterior olfactory nucleus, CoA, Cortical amygdala, CV, Coefficient of variation, DPC, Dorsal peduncular cortex, DTT, Dorsal tenia tecta, EC, Entorhinal cortex, ISI, Interspike interval, OB, Main olfactory bulb, OT, Olfactory tubercle, PC, Piriform cortex, TT, Tenia tecta, VTT, Ventral tenia tecta, Olfaction, olfactory peduncle, tenia tecta, anterior olfactory nucleus, sensory tuning

## Abstract

The tenia tecta is extensively interconnected with the main olfactory bulb and olfactory cortical areas and is well positioned to contribute to olfactory processing. However, little is known about odor representation within its dorsal (DTT) and ventral (VTT) components. To address this need, spontaneous and odor-evoked activity of DTT and VTT neurons was recorded from urethane anesthetized mice and compared to activity recorded from adjacent areas within adjacent caudomedial aspects of the anterior olfactory nucleus (AON). Neurons recorded from DTT, VTT, and AON exhibited odor-selective alterations in firing rate in response to a diverse set of monomolecular odorants. While DTT and AON neurons exhibited similar tuning breadth, selectivity, and response topography, the proportion of odor-selective neurons was substantially higher in the DTT. These findings provide evidence that the tenia tecta may contribute to the encoding of specific stimulus attributes. Further work is needed to fully characterize functional organization of the tenia tecta and its contribution to sensory representation and utilization.

## Introduction

Mitral and tufted cells of the main olfactory bulb (OB) innervate numerous diverse, interconnected structures within the ventral telencephalon. Collectively, these areas decode spatiotemporal patterns of OB glomerular activity and contribute to odor perception and odor-guided biobehavioral processes. Variation in the spatial topography of OB projections, as well as heterogeneity in the cellular composition and local circuit organization of bulbar targets, provide the architecture which may support diversity in sensory representation and utilization across olfactory areas (Giessel & Datta, 2014; Sosulski et al., 2011).

Sensory physiological studies characterizing principles of sensory representation are necessary to provide insight to fully develop theories addressing functional diversity within the olfactory system. Although piriform cortex (PC), the most prominent OB target, has been the main focus for physiological investigation among primary olfactory areas (Isaacson, 2010; Stettler & Axel, 2009; Wilson & Sullivan, 2011), a growing body of research is examining features of sensory coding and functional organization in closely associated olfactory regions, including the olfactory tubercle (OT) (Gadziola et al., 2015; Payton et al., 2012; Rampin et al., 2012; Wesson & Wilson, 2010), entorhinal cortex (EC) (Xu & Wilson, 2012), cortical amygdala (CoA) (Iurilli & Datta, 2017), and the anterior olfactory nucleus (AON) (Kikuta et al., 2008, 2010; Lei et al., 2006; ; Tsuji et al., 2019). Limited data available to date from single-unit electrophysiological studies suggest that, despite cytoarchitectural and organizational differences across olfactory areas, populations of neurons often exhibit response characteristics that resemble those observed in PC (Iurilli & Datta, 2017; Payton et al., 2012).

The cortical structures lining the medial wall of the olfactory peduncle are extensively interconnected with the OB and other primary olfactory structures and are prominently positioned to contribute to olfactory processing (Brunjes, 2011). These areas, which includes the dorsal and ventral tenia tecta (DTT, VTT) and dorsal peduncular cortex (DPC), have been differentiated within olfactory peduncle from the AON and from more lateral olfactory cortical areas, including PC, OT, and EC (Ennis et al., 2015; Haberly, 2001; Shipley et al., 2004), though they exhibit the trilaminar organization typical of primary olfactory cortex (Haberly & Price, 1978). Despite the likely role of medial peduncular cortical areas in olfactory information processing, physiological studies are sparse, and little is known about odor representation within these areas or their functional organization.

In an initial attempt to investigate potential functional diversity within the olfactory peduncle, single-unit extracellular recordings were conducted in urethane-anesthetized mice, and spiking activity was evaluated in response to a range of monomolecular odorants. Recordings targeted the DTT and VTT, which form a narrow, mostly vertically-oriented strip of cortex with well-defined laminae, and the activity of recorded cells was compared to that of more laterally situated cells inadvertently recorded from caudomedial components of the AON. Cells in DTT, VTT, and AON samples often fired in relation to ongoing respiration with similar peak phase and modulation depth. Although olfactory response properties were similar between regions, the proportion of odor-selective neurons was greater in the DTT.

## Experimental procedures

### Subjects

Adult C57BL/6 mice bred at Drew University and aged eight to 20 weeks were used in the present study. Subjects were housed in same-sex cohorts in clear plastic cages in a temperature- and humidity-controlled vivarium and were maintained on a reversed 12-hour light/dark cycle with *ad libitum* access to standard laboratory chow and water. Experiments were conducted in accordance with the National Institute of Health Guide for the Care and Use of Laboratory Animals (NIH Publications No. 80-23), and procedures were approved by the Drew University Institutional Animal Care and Use Committee.

### Surgery

Surgical procedures were conducted to permit dorsoventral advancement of recording electrodes targeting the medial wall of the olfactory peduncle in the left hemisphere. Subjects were anesthetized with urethane (target dose 1.5 mg/kg, IP), supplemented with atropine (25 mg/kg, IM) to reduce respiratory secretion, and were mounted in a standard stereotaxic frame. During surgery the level of anesthesia was assessed by monitoring respiration rate and hindlimb withdrawal, and isoflurane was administered acutely (INH) as needed. The skull surface was exposed, and a PFA-coated stainless-steel reference wire (127 μm bare diameter; A-M Systems, Sequim, WA) was placed in the right parietal lobe through a small craniotomy. A stainless-steel head plate (Model 10, Neurotar, Helsinki, FIN) was affixed to the skull with dental acrylic, and craniotomy was performed through the aperture, centered at midline and 2.25 mm anterior to bregma.

### Electrophysiological recording

Following surgery, subjects were positioned in a headplate clamp affixed to an anti-vibration platform. Recordings were conducted within a Faraday cage situated within a laboratory fume hood in order to minimize electrical noise and to rapidly remove odorized air. A tungsten microelectrode (12 MOhm, A-M Systems, Sequim, WA) was advanced ventrally using a Huxley-style micromanipulator (MX310, Siskiyou, Grants Pass, OR), and recordings commenced as soon as sufficiently large spike waveforms were maintained stably for at least 5 minutes at depths greater than 2.5 mm. In cases where two or more recording sites occurred in the same electrode pass, the electrode was advanced until the absence of spikes from the initial recording was confirmed by inspection of oscilloscope traces. Neural signals were amplified and filtered (10,000X, 500 Hz - 3 kHz; A-M Systems Models 1800 or 3600), and digitized (20 kHz; POWER 1401, Cambridge Electronic Design, Cambridge, UK) for storage and analysis. For most recordings, respiration was monitored throughout the session using a piezoelectric sensor positioned under the chest.

### Stimulus presentation

Following the recording of stable baseline firing rates for a minimum 5 min period, a sequence of monomolecular odorants (2 sec duration, 28 sec interstimulus interval; median 8 trials per odorant) was presented using a custom-built flow-dilution olfactometer controlled by Spike2 software (Cambridge Electronic Design). Five to 13 odorants (median 11 odorants) were selected from the following: 1,7-octadiene, propyl butyrate, 2-heptanone, isoamyl acetate, heptanal, limonene, 5-methyl-2-hexanone, ethyl valerate, nonane, 1-pentanol, 4-methyl-3-penten-2-one, 3-methyl-2-buten-1-ol, and 1,8-cineole. All odorants were diluted in mineral oil to reach 100 ppm in the vial headspace using methods described by Barnes et al. (2008) and were delivered in oxygen gas through Teflon tubing at 1.0 l/min. One additional channel was reserved for mineral oil alone as a control stimulus. In order to minimize possible effects odor source position on neuronal firing (Kikuta et al., 2008, 2010;) odor tubes were fed through a manifold with a single outlet port oriented rostrocaudally and positioned 2 cm from the nares. The manifold was cleaned daily in order to minimize stimulus contamination.

### Identification of recording sites

Following data collection, brains were extracted and fixed in 10% formalin solution for 10-14 days. Tissue was embedded in egg yolk, sectioned at 50 μm, and mounted onto gelatin-coated microscope slides. Recording sites were identified in Nissl-stained sections as the ventral-most extent of damage caused by electrode passage or estimated based on logged recording depth within histologically verified tracks in cases where recordings were attempted at two or more depths within the same electrode pass. In six Ss, recordings were made in more than one electrode pass, and electrode tracks were differentiated based on logged anteroposterior and mediolateral electrode positions. Anatomical classification of each recording site was based on the atlas of Paxinos & Franklin, 2019.

### Data Analysis

Spike waveforms were extracted and isolated using OfflineSorter (Plexon, Inc., Dallas, TX), and single units were verified based on waveform shape, waveform parameter clusters, and interspike interval histograms. All other analyses were conducted using custom scripts written in Matlab (The Mathworks, Natick, MA). Each cell’s session-wide spontaneous firing rate was estimated as the reciprocal of the mean interspike interval (ISI) for all spikes occuring during the final 10 sec period of the insterstimulus interval, after the evacuation of detectable odor. Patterns of spontaneous spiking output (e.g., bursting activity) were assessed by calculating coefficient of variation (CV) values for each cell’s ISI distribution (ISI SD / ISI mean) during the same period. Statistical comparison of spontaneous activity metrics between regions was performed using Mann-Whitney tests.

The effect of odor presentation on neural activity was assessed by comparing spike counts observed during the 4 sec interval immediately following odor onset (odor period) to that of the immediately preceding 4 sec interval (baseline period). Given that neural firing rates typically exhibit log-normal distributions (Buzsaki, 2005), effect sizes of odor-evoked firing rate changes were first estimated using a non-parametric rank-biserial correlation (Cureton, 1956) for each odor-cell pair. However, a substantial proportion of calculated correlations (130/973 total) were perfect (*r* = 1.0), which constrained effect size estimates, so odor period firing rates were instead expressed as z-scores relative to the baseline mean rate for each pair ([odor period firing rate - baseline period firing rate] / baseline period SD). The extent of odor selectivity was assessed for each cell by calculating a selectivity index (Meyer et al., 2011), [(maxrate – minrate) / (maxrate + minrate)], where in this case maxrate and minrate refer to maximum and minimum mean odor period firing rates across all presented odorants (omitting the vehicle control). Statistical significance of odor-evoked firing rate alterations was assessed for each odor-cell pair using Wilcoxon signed-rank tests for paired samples, and interregional comparisons in neural activity metrics were performed using Mann-Whitney tests.

Rhythmic modulation of spiking activity in relation to ongoing respiration was evaluated using methods developed by (Drew & Doucet, 1991) and applied to respiration phase by (Litaudon et al., 2003). Briefly, the variable duration respiration cycles were converted to degrees (0/360 deg. = inhalation-exhalation transition), and spikes were assigned proportional phase values relative to inspiration onset for each respiratory cycle. Phase values were binned in 6-degree intervals to create a distribution of spike phases, and the summed counts for each bin were converted to vectors, each representing the magnitude of a cell’s response at a particular respiration phase. The angle (theta) and magnitude (rho) of each cell’s resulting population vector was used as an index of preferred phase and the overall magnitude of firing rate respiratory modulation, respectively. Significant deviation in the distribution of spikes across respiratory cycles was evaluated using Rayleigh tests, and all cells with significant directional means were confirmed to have unimodal circular distributions.

## Results

**Fig. 1:**
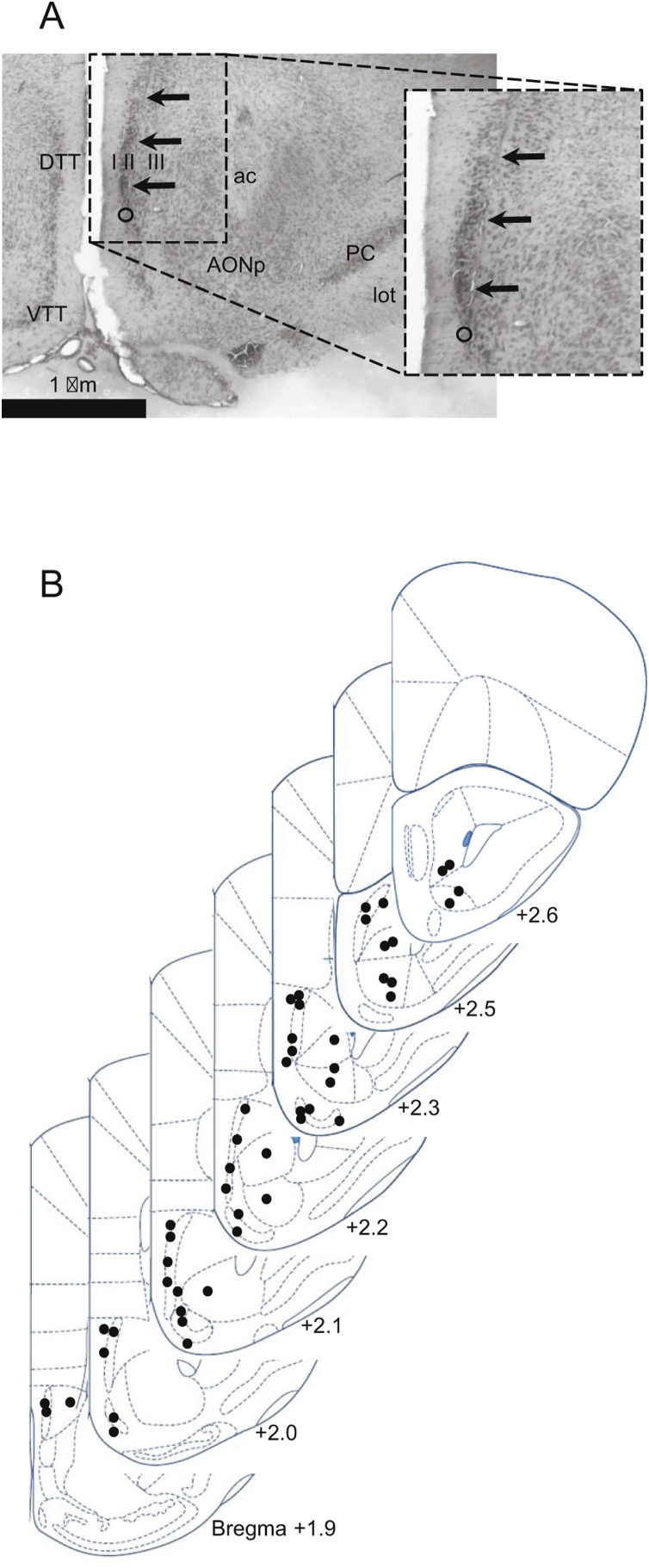
**Extracellular recording from TT and AON.** (A) Recording site identified in DTT Layer II in a Nissl-stained section. DTT, dorsal tenia tecta (layers I, II, III); VTT, ventral tenia tecta; AONp, anterior olfactory nucleus, pars posterioralis; PC, piriform cortex; lot, lateral olfactory tract; ac, anterior commissure. Arrows denote electrode track; circle denotes recording site. (B) Coronal sections showing the estimated location of recording sites (filled circles) within medial olfactory structures. Sites within DTT, VTT, and AON were included in analyses. Figures were adapted from (Paxinos & Franklin, 2019).

A total of 89 cells isolated from 50 recording sites in 21 subjects (9 female; 12 male) were analyzed in the present study, and data files for all cells have been made available at a published repository (Cousens, 2020). Thirty-seven cells (23 female; 14 male) were recorded from the DTT, and 15 cells (6 female; 9 male) were recorded from the VTT. Thirty-seven cells (9 female; 28 male) were recorded from AON pars principalis. Given the lack of clear anatomical boundaries between subregions within AON pars principalis, cells recorded from AON pars medialis, pars posterioralis, and pars ventroposterioralis were combined into a single sample. Due to the relatively small size of the VTT sample, primary inferential analyses comparing activity between samples were limited to the DTT and AON. However, since limited published data is available on the response properties of VTT neurons, descriptive data on the VTT spontaneous and odor-evoked activity was provided where appropriate. Fig. 1 illustrates the estimated locations of recording sites for the sample of analyzed cells. Recordings were restricted to the olfactory peduncle, where DTT and VTT co-occur within the same coronal plane, and no recordings were obtained from the DTT caudal to Bregma +1.75 mm. Additional recording sites were identified in adjacent regions, including the OB, the OT, the lateral septal nucleus, and the nucleus accumbens shell, but were not included in the analyses.

Evaluation of sex differences in spontaneous activity was conducted on the full sample due to unbalanced distribution of sex across the three regions. Neither spontaneous firing rates (median: female, 1.55 Hz; male, 1.26 Hz; *U* = 909.5; *n* = 89; *p* = 0.622) nor ISI CV values (median: female, 1.01; male, 1.08; *U* = 1006; *n* = 89; *p* = 0.759) differed between sexes. No gross differences in spontaneous activity were observed between the DTT and AON samples. Spontaneous firing rates were similar between regions (median: DTT, 1.51 Hz; AON, 1.25 Hz; *U* = 723.5; *n* = 74; *p* = 0.673) as were ISI CV values (median: DTT, 1.06; AON, 1.10; *U* = 682; *n* = 74; *p* = 0.978).

**Fig. 2:**
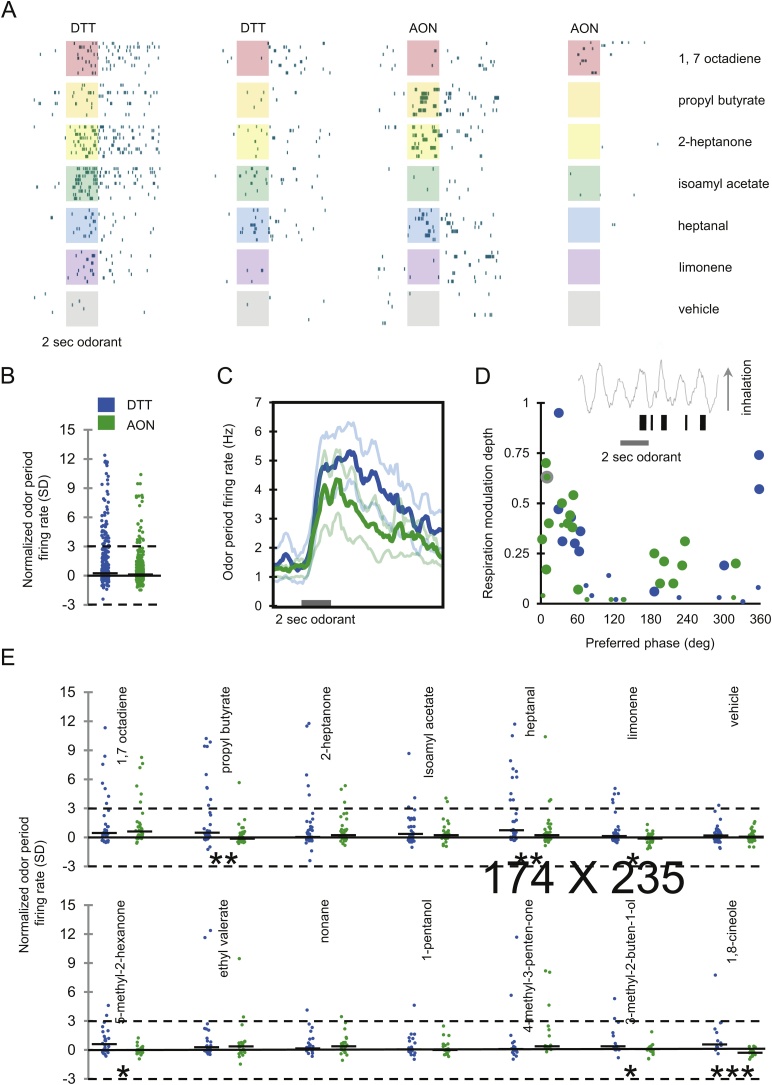
**Odor-evoked spiking activity in DTT and AON.** (A). Raster plots showing sample spiking activity of two DTT neurons (left) and two AON (right) neurons in response to presentation of six monomolecular odorants sampled from up to 13 per session plus mineral oil vehicle. Shaded areas indicate odor presentation (2 sec) with 8-9 trials per odorant presented in pseudorandom sequence. (B) Normalized odor-period firing rates for all odor-cell pairs. Firing rates during the 4 sec period beginning with odor onset are expressed as z-scores relative to the mean pre-odor baseline firing rate (see Experimental Procedures). DTT, *n* = 401; AON, *n* = 410. (C) Grand average peristimulus time histograms for odor-cell pairs with odor-period firing rates > 3 SD above pre-odor baseline. Traces show mean response rate ± SEM. DTT, *n* = 55; AON, *n* = 22. (D) Relationship between preferred respiration phase and degree of respiration-related firing rate modulation (see Experimental Procedures) during the 8 sec period beginning with odor onset across all cells recorded with respiration signals (DTT, *n* = 21; AON, *n* = 26). Large symbols indicate cells with significant unimodal deviations from a uniform phase distribution (Rayliegh test, *p* < 0.01). **(**Inset) Raw spiking activity from an example TT neuron and waveform of ongoing respiration cycles surrounding odor presentation. (E) Normalized odor-period firing rates for all odor-cell pairs segregated according to odorant. Mann-Whitney tests conducted between TT and AON samples: * *p* < .05; ** *p* < .01; *** *p* < .001.

Presentation of monomolecular odorants resulted in phasic alterations in firing rate in cells recorded from the DTT and the AON. As illustrated in Fig. 2, responses varied in onset latency, duration, magnitude, and tuning breadth, but the range of response topographies were similar across samples. Observed alterations in firing rate typically occurred during the first or second respiratory cycle after odor onset and remained elevated for several seconds after odor offset (Fig. 2A, C).

A total of 811 odor-cell pairs were recorded from the DTT (401 pairs) and the AON (410 pairs). To quantify odor-evoked responses, odor period firing rates were expressed as z-scores relative to baseline (see Experimental Procedures). Most responses were tightly distributed around zero (median: DTT, 0.333; AON, 0.104), indicating minimal firing rate change in response to odor onset (Fig. 2B, E). However, odor-evoked alterations were observed in a substantial proportion of responses (≥3 SD from baseline: DTT, 14.1%; AON, 5.5%), and all of these responses were excitatory. The proportion of odor-cell pairs (Wilcoxon signed-rank test, *p* < 0.01) with significantly elevated odor period firing was greater in the DTT sample (43/401 pairs, 10.7%) than the in AON sample (11/410 pairs, 2.7%), and the proportion of cells significantly responsive to any odorant was also greater in the DTT sample (11/37 cells, 29.7%) than in the AON sample (4/37 cells, 10.8%). Despite these differences in odor responsivity, peri-stimulus time histograms showing firing rates averaged across all excitatory odor-cell pairs (≥ 3 SD from baseline) revealed that the overall time course and topography of odor-evoked responses were similar in the two samples (Fig. 2C).

The overall pattern of responsivity across the full panel of odorants was similar between the two samples (Fig. 2E). Significant alterations in firing rate were not found in response to vehicle in either sample, and neither sample appeared to show any preference for odorants from any particular chemical class or functional group. However, consistent with greater overall responsivity in the DTT, normalized response rates were significantly higher for DTT cells compared to AON cells for six of the 13 odorants (Mann-Whitney tests, p < 0.05), while the reverse was not true for any odorant. Further, although significant excitatory responses were observed in both samples, the proportion of responsive cells was greater in the DTT for most odorants (DTT > AON: 9/13 odorants; AON > DTT: 0/13 odorants). Among responsive neurons in both samples, the majority responded to more than one odorant (DTT, 9/11; AON, 4/4), and the median number of odorants to which responsive cells exhibited significant excitation was similar (DTT, 4/13; AON, 2.5/13). The selectivity index assessed across all odorants did not differ between full samples of DTT and AON cells (median: DTT, 1.18; AON, 1.39; *U* = 612.5; *n* = 74; *p* = 0.436) or between samples of responsive cells (median: DTT, 1.05; AON, 1.13; *U* = 10.0; *n* = 15; *p* = .138), indicating that odor period firing rates in the two regions differentiated odorants to a similar extent. Collectively, these findings demonstrate that neurons within both the DTT and the AON are responsive to presentation of monomolecular odorants in the anesthetized mouse but that a higher proportion of DTT neurons are responsive and may exhibit increased sensitivity to olfactory stimulation compared to those recorded from the AON. Further, although most neurons were excited by multiple stimuli, responsive cells were generally selective and responded to a limited subset of presented odorants.

Neural activity was assessed in relation to ongoing respiration during 8 sec intervals beginning at odor onset in the subset of cells for which respiration signals were recorded (DTT, *n* = 21; AON, *n* = 26). A majority of cells in both samples fired in phase with ongoing respiration, with the deepest respiration-related modulation occurring just after the inhalation-exhalation transition (Fig. 2D). The percentage of cells exhibiting significant respiratory modulation (Rayleigh test; *p* < 0.01) was slightly greater in the AON sample (18/26 cells, 69.2%) than in the DTT sample (11/21 cells, 52.6%). However, the distributions of phase preferences across the two samples were similar, as were the mean phase preferences estimated by the vector sum of all modulated cells within each sample (DTT, 26.7 deg; AON, 21.6 deg).

Given substantial differences in cytoarchitectural and connectional features between the VTT and DTT, it was of interest to examine the response properties of VTT neurons despite the relatively small sample size. Fifteen cells were judged to have been recorded from the VTT, contributing 162/973 (16.6%) of total odor-cell pairs reported in the study. Spontaneous firing rates (median: VTT, 2.01 Hz) and ISI CV values (median: VTT, 0.75) observed in the VTT sample were similar to those observed in the DTT sample. However, the proportion of odor-cell pairs with significantly elevated odor period activity (Wilcoxon signed-rank test, p < 0.01; 3/162 pairs, 1.9%) was substantially lower than that observed in the DTT, as was the proportion of cells significantly responsive to any odorant (2/15 cells, 13.3%). Further, the proportion of VTT cells exhibiting significant respiratory modulation (Rayleigh test; *p* < 0.01; 2/15 cells, 13.3%) was lower than that observed in the DTT. These findings demonstrate that VTT neurons exhibit odor-selective excitation but that the VTT may be less responsive to olfactory stimulation compared to the DTT. However, this conclusion must be tempered by the limited size of the VTT sample.

## Discussion

The present study characterized spontaneous and odor-evoked spiking activity of cells within the medial wall of the olfactory peduncle in the anesthetized mouse. Cells recorded from the DTT, VTT, and caudomedial aspects of the AON exhibited odor-selective alterations in firing rate in response to a diverse array of monomolecular odorants and often fired in relation to ongoing respiration. Neural activity recorded from the DTT was directly compared to that recorded from the AON, and despite similar tuning breadth, selectivity, and response topography between samples, a greater proportion of DTT neurons exhibited odor-evoked excitation. In contrast, the spiking activity of AON neurons was more likely to be modulated by ongoing respiration compared to that recorded from DTT neurons.

This study provides among the first electrophysiological evidence for tuned odor-evoked activity within the tenia tecta. Recent work by Shiotani et al. (2018) conducted in mice performing a go/no-go olfactory discrimination task revealed that roughly a quarter of VTT neurons exhibited maximal firing during odor sampling. However, as indicated by the authors, given that the majority of these cells showed rate modulation prior to odor onset, the extent to which such activity was driven by olfactory input was unclear. The paucity of additional relevant electrophysiological research limits conceptual advancement in understanding of the contributions of the tenia tecta to olfactory processing.

The relative robustness of odor evoked activity observed here in the DTT is consistent with some degree of functional heterogeneity between the dorsal and ventral components. The VTT is a trilaminar structure with a well-developed pyramidal cell layer typical of primary olfactory cortex in terms of organization, cytoarchitecture, and connectivity and establishes reciprocal connections with the OB, as well as PC, EC and AON pars principalis (Haberly & Price, 1978). In contrast the DTT, which forms a conspicuous, semi-continuous band extending caudally from the OB to the genu of the corpus callosum and continuing along the dorsal surface of the callosum as the induseum griseum, is arguably more closely associated with the hippocampus than with olfactory cortex (Haberly & Price, 1978; Wyss, 1981; Wyss & Sripanidkulchai, 1983). The DTT receives input from PC and lateral EC but comparatively weak input from the OB, and feedback from the DTT to the OB and olfactory cortical structures is weak. Thus, the finding here that odor-evoked activity within the DTT may be substantially more robust than that of the VTT is surprising. However, the lateral EC may be one source of feed-forward olfactory input to the DTT. Using methods similar to those described here, Xu and Wilson (2012) reported that lateral EC units exhibited odor-evoked excitation, though EC cells were less responsive and more narrowly tuned compared to those recorded from anterior PC. Approximately 20% of EC units fired in phase with ongoing respiration, similar to that observed here in the DTT and significantly lower than that observed in anterior PC (Xu & Wilson, 2012), which more closely resembled the present sample of AON cells with a high degree of respiration-entrained activity. Given relatively weak input to the DTT from the OB, it is tempting to speculate that odor representation within the TT may depend on input from EC and PC.

The present findings confirm that cells recorded from AON pars principalis exhibit odor-specific alterations in firing rate. Early work by Boulet et al. (1978) demonstrated that cells recorded from ventral AON in the rabbit were more broadly tuned than OB mitral cells and that, in contrast to the present findings, often exhibited inhibitory responses to odor presentation. Lei et al. (2006) demonstrated that histologically identified pyramidal neurons intracellularly recorded from the AON were broadly tuned to multiple components of odor mixtures, a pattern that may emerge through convergence of synaptic input from narrowly-tuned OB mitral cells (Apicella et al., 2010; Miyamichi et al., 2011) and through integration of OB input through associational fiber networks (Luskin & Price, 1983). Tsuji et al. (2019) recently reported evidence for several classes of rhythmic cells in the ventrolateral AON in urethane anesthetized rats. Despite a higher median spontaneous rate compared to that observed in the present sample, a similar proportion of cells were responsive to odor presentation. Consistent with these findings, the majority of cells assessed fired in phased with ongoing respiration with a preference for peak firing during the expiration phase, which may relate in part to GABAergic inhibition resulting from activation of the lateral olfactory tract (Tsuji et al., 2019).

The AON is a relatively simple cortical structure comprised of two primary regions (Brunjes et al., 2005; Brunjes & Illig, 2008). Pars principalis is a thick two-layered cortical structure reciprocally connected with both the OB and PC and comprised of a variety of cell types (Kay & Brunjes, 2014). It occupies a prominent position within the olfactory peduncle and is encapsulated in some planes by pars externa, a thin strip of large cells lacking basal dendrites. Detailed physiological examination of the functional organization of pars principalis has not been carried out to date. However, evidence showing spatially distributed patterns of Fos-immunolabeling following odorant exposure (Kay et al., 2011) suggest a lack of fine spatial topography, similar to that observed in PC (Stettler & Axel, 2009) and differing markedly from the highly organized structure of AON pars externa (Grobman et al., 2018; Schoenfeld & Macrides, 1984; Scott et al., 1985; Yan et al., 2008). The AON has long been proposed to play a prominent role in feedback regulation of OB activity, and projections from the AON pars principalis to the OB show variation in bilateral symmetry, fiber segregation, and terminal distribution within the OB lamina (Davis & Macrides, 1981; Illig, 2011). Optogenetic stimulation of AON fibers projecting to the OB was found to exert direct synaptic effects on both OB interneurons and mitral cells, inhibiting mitral cell activity overall and enhancing the temporal precision of odor-evoked spiking (Markopoulos et al., 2012). Thus, in conjunction with corticofugal projections from PC, feedback projections from the AON play functional role in sculpting patterns of OB activity (Boyd et al., 2012; Otazu et al., 2015; Rothermel & Wachowiak, 2014), presumably influencing OB output to wide expanses of olfactory cortex. Consistent with this role, evidence suggests that manipulation of the activity of OB-projecting neurons within AON pars medialis bidirectionally modulates olfactory sensitivity in behaving mice (Aqrabawi et al., 2016).

The recording methods utilized in the present study present several limitations which must be addressed. Given the organization of medial olfactory cortex and that the AON pars medialis and pars ventroposterioralis extend toward the pial surface in some coronal planes, where they interdigitate with the tenia tecta, the classification of recordings in the present study must be considered a first-order scheme. Multiple recordings were often made within the same electrode track and recording sites were estimated based on logged recording depth, which produces some degree of uncertainty in the precision of recording site placement. Further, the extracellular recording methods utilized here preclude the identification of recorded cells, which limits speculation about the role of particular cell types in information processing. Indeed, considerable heterogeneity exists in cell morphology within the DTT, VTT, and AON (Kay & Brunjes, 2014; McGinley & Westbrook, 2010; Wyss & Sripanidkulchai, 1983), and sophisticated accounts of the role of these areas in olfactory processing must take into consideration cellular diversity. High density electrophysiological recording (Iurilli & Datta, 2017) or optical recording methods (Stettler & Axel, 2009) enabling larger sample sizes and better estimation of the spatial characteristics of recordings would facilitate a more detailed analysis of functional organization of the region and comparison of inter- and intra-regional differences in response characteristics. Additionally, recordings were conducted here in urethane anesthetized mice in order to characterize the sensory properties of TT neurons and to minimize the impact of neural activity associated with ongoing behavior. However, it is of interest to characterize tenia tecta activity during active exploration in awake, behaving animals given the dominance of oscillatory activity associated with active sniffing in modulating spiking activity in the OB and olfactory cortex (Eeckman & Freeman, 1990; Frederick et al., 2016).

To summarize, the present study characterized spontaneous and odor-evoked activity within the DTT, VTT, and caudomedial aspects of the AON in the anesthetized mouse and demonstrated that cells within all three regions exhibit odor-selective alterations in firing rate in response to a diverse array of monomolecular odorants. Further, cells in each region often fire in phase with ongoing respiration, consistent with extensive interconnectivity with other primary olfactory cortical areas and the OB. The finding that odor-evoked activity was particularly robust in the DTT is a novel finding and suggests that this region may play a prominent role in olfactory information processing and in mediating communication between olfactory structures and the hippocampal formation.

## Conflicts of interest

None.

## Data Repository

https://github.com/grahamcousens/characterization-of-odor-evoked-neural-activity-in-the-olfactory-peduncle.
